# Causal Hierarchy within the Thalamo-Cortical Network in Spike and Wave Discharges

**DOI:** 10.1371/journal.pone.0006475

**Published:** 2009-08-03

**Authors:** Anna E. Vaudano, Helmut Laufs, Stefan J. Kiebel, David W. Carmichael, Khalid Hamandi, Maxime Guye, Rachel Thornton, Roman Rodionov, Karl J. Friston, John S. Duncan, Louis Lemieux

**Affiliations:** 1 Department of Neurology, Policlinico Umberto I°, University of Rome “La Sapienza”, Rome, Italy; 2 Department of Clinical and Experimental Epilepsy, UCL Institute of Neurology, London, United Kingdom; 3 MRI Unit, National Society for Epilepsy, Chalfont St. Peter, United Kingdom; 4 Wellcome Trust Centre for Neuroimaging, UCL, London, United Kingdom; 5 Department of Neurology and Brain Imaging Center, Johann Wolfgang Goethe-University, Frankfurt am Main, Germany; 6 CRMBM, CNRS UMR6612 & INSERM, Unit ‘Epilepsy and Cognition’ U751, CHU TIMONE et Université de la Méditerranée, Marseille, France; 7 Max Planck Institute for Human Cognitive and Brain Sciences, Leipzig, Germany; Instituto Cajal - CSIC, Spain

## Abstract

**Background:**

Generalised spike wave (GSW) discharges are the electroencephalographic (EEG) hallmark of absence seizures, clinically characterised by a transitory interruption of ongoing activities and impaired consciousness, occurring during states of reduced awareness. Several theories have been proposed to explain the pathophysiology of GSW discharges and the role of thalamus and cortex as generators. In this work we extend the existing theories by hypothesizing a role for the precuneus, a brain region neglected in previous works on GSW generation but already known to be linked to consciousness and awareness. We analysed fMRI data using dynamic causal modelling (DCM) to investigate the effective connectivity between precuneus, thalamus and prefrontal cortex in patients with GSW discharges.

**Methodology and Principal Findings:**

We analysed fMRI data from seven patients affected by Idiopathic Generalized Epilepsy (IGE) with frequent GSW discharges and significant GSW-correlated haemodynamic signal changes in the thalamus, the prefrontal cortex and the precuneus. Using DCM we assessed their effective connectivity, i.e. which region drives another region. Three dynamic causal models were constructed: GSW was modelled as autonomous input to the thalamus (model A), ventromedial prefrontal cortex (model B), and precuneus (model C). Bayesian model comparison revealed Model C (GSW as autonomous input to precuneus), to be the best in 5 patients while model A prevailed in two cases. At the group level model C dominated and at the population-level the p value of model C was ∼1.

**Conclusion:**

Our results provide strong evidence that activity in the precuneus gates GSW discharges in the thalamo-(fronto) cortical network. This study is the first demonstration of a causal link between haemodynamic changes in the precuneus - an index of awareness - and the occurrence of pathological discharges in epilepsy.

## Introduction

The existence of a link between physiological and environmental factors and the occurrence of epileptic seizures is well documented in the literature [1]–[4]. However, the brain networks through which these factors influence the epileptic state remain unclear.

Such a link is particularly evident in patients affected by Idiopathic Generalized Epilepsy (IGE) in whom, for example, sleep deprivation, alcohol and stress seem to act as activating factors for seizures occurrence [3] and a close relationship between absences and the sleep-wake cycle has been demonstrated [1]–[3].

The prototypical seizure type in IGE is the absence with its electroencephalographic hallmark, generalised spike and wave (GSW) discharges. Clinically, absences are characterized by a blank stare and impaired consciousness. Activities requiring vigilant attention have been coupled with a lesser likelihood of absences whereas an increased frequency of these seizures during relaxation is well established [3], [4]. These findings suggest a causal link between changes in the level of awareness and the occurrence of GSW discharges.

Recent functional imaging studies have revealed the existence of a set of brain regions which show increased functional and metabolic activity during rest, compared to attention-demanding tasks [5], [6]. Involved brain areas include the posterior cingulate cortex, the precuneus, the medial prefrontal cortex, mid-dorsolateral prefrontal and anterior temporal cortices and have been hypothesized to constitute the so-called “default mode network” (DMN) [5]. DMN activity decreases during various cognitive tasks indicate that this network sustains the spontaneous thought processes or self-oriented mental activity that characterizes the brain's resting state. Mental processes subservient to consciousness have been linked to DMN activity such as random episodic memory [7], conceptual processing [8], stimulus independent thought [9] and self-reflection [10]–[13]. Although most neuroimaging studies characterize the DMN as a homogeneous network, recent work has suggested a functional differentiation within it: particularly, of the two main nodes in the DMN, the posteromedial cortical region (precuneus and posterior cingulate cortex) seem linked specifically with visual-spatial and attention networks while the medial prefrontal cortex is more engaged in motor control circuits [14]. Additionally, the precuneus/posterior cingulate node has been recently demonstrated to have the highest degree of interactions (using a partial correlation approach on fMRI data) with the rest of the DMN [15] suggesting a pivotal role of this area within the network. This interpretation is in line with evidence from previous PET studies that this brain region, and in particular the precuneus, shows the highest metabolic rate consuming 35% more glucose than any other area of the cerebral cortex [16] at rest.

The DMN shows decreased activity both during attention-demanding tasks and equally during states of reduced vigilance and, especially the posteromedial cortical regions, during altered states of consciousness [17], [18]. Based on these observations, several authors [12], [13], [19] suggested a pivotal role of the posteromedial cortical region in self-consciousness inside the DMN.

EEG-correlated functional magnetic resonance imaging (EEG-fMRI) studies have shown a common pattern of blood oxygen level-dependent (BOLD) signal decrease in the precuneus and the other default mode areas, together with a thalamic BOLD signal increase, during ictal and interictal GSW discharges [20]–[27]. Decreased cerebral blood flow consistent with a decrease in neuronal activity was demonstrated in DMN regions during GSW [28]. Therefore, these relative BOLD signal decreases can be interpreted as a transitory suspension of the “default state” of the brain which occurs in association with an altered level of awareness observed during GSW discharges and absences, respectively [22]–[25].

The pathophysiological substrate of GSW remains enigmatic and several studies, both in animals and humans, have tried to answer the historical debate regarding the putative role of the thalamus and cortex as generators. Data from invasive recordings and manipulations in well-validated genetic models of absence epilepsy have supported the hypothesis that absence seizures are of cortical origin. Depth electrode recordings from the thalamus and suprasylvian cortex in cats have shown a primary role of the neocortex in producing seizures consisting of spike and wave complexes [29]. More recently, electrophysiological recordings in a rat (WAG/Rij) genetic model of absence epilepsy demonstrated the existence of a cortical focus within the perioral regions of somatosensory cortex [30], [31]. Using *in vivo* intracellular recordings in the GAERS rat model (Genetic Absence Epilepsy Rats from Strasbourg), Polack and colleagues [32] demonstrated pathological activity originating in the perioral region of somatosensory cortex. Those findings led to renewed interest in the role of the cortex in human GSW. Using source reconstruction methods based on high-density EEG data acquired during the propagation of ictal GSW discharges, Holmes et al. [33] showed the involvement of the orbital frontal and mesial frontal regions. Recent work using advanced EEG data analysis provided evidence in favour of a primary role of ventromedial prefrontal cortex (vmPFC) and particularly Brodmann area 10 in the generation of GSW discharges during absences [34].

However, despite the suggestion of the involvement of dorsal cortical regions (particularly posterior-medial cortical regions) in GSW discharges from neuroimaging studies [22]–[25] and their role in conscious awareness [18], [19], no work attempting to understand the interaction between these areas and the (frontal)cortical-thalamic loop has been performed to date. This is what we propose to evaluate here using Dynamic Causal Modelling to study effective connectivity based on simultaneously recorded EEG-fMRI data in patients with GSW discharges.

Dynamic Causal Modelling (DCM) offers the possibility of characterising the effective connectivity, defined as “the influence that one neural system exerts over another”, in other words: it can be used to test which brain region drives which [35]. The aim of DCM is to estimate the effective connectivity between brain regions and more generally, to adjudicate among a set of models describing connectivity using model comparison [36]. In brief, DCM for fMRI data combines a model of neural dynamics with an experimentally validated haemodynamic model that describes the transformation of neuronal activity into a BOLD response [37]. Both sets of parameters describing the neuronal state and those determining the forward model of BOLD signal generation [36] are estimated from the data within a Bayesian framework for each brain area included in the model. Hence, crucially, the possibility for differing haemodynamic responses (e.g. latency between regions) is included within the DCM. The Bayesian framework allows an inference to be made as to whether the data is best explained by variations in the haemodynamic response or instead by changes in the underlying neural system. This means valid inferences can be made about, for example, which brain region drives which, despite the limitations of temporal resolution inherent to fMRI. This could be particularly interesting in epilepsy where identifying the drivers of the pathological activity and their “causal” relationships in the epileptic network is essential for improving neurophysiopathological understanding of epileptic syndromes. Hamandi et al. [38] used DCM to show propagation of neuronal activity from the irritative zone to ipsilateral posterior brain regions in a patient with temporal lobe epilepsy. More recently David and colleagues [39] applied effective connectivity analysis techniques including DCM to fMRI data from (GAERS) rats to demonstrate concordance between the drivers revealed by DCM and the trigger identified using intracranial recordings.

**Figure 1:**
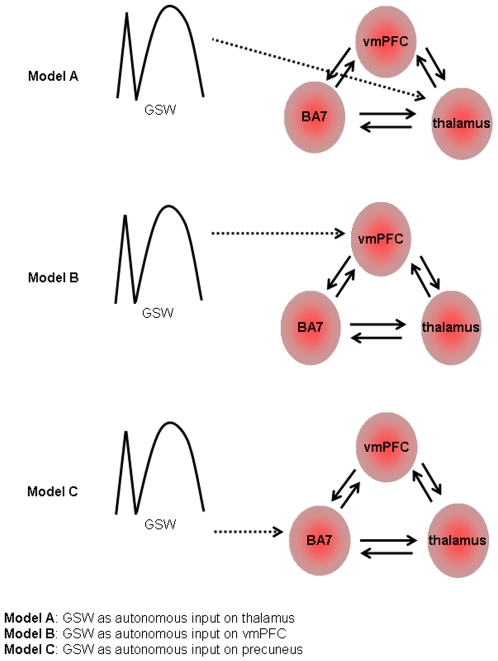
Effective connectivity models. Effective connectivity (DCM) models showing GSW discharges as autonomous input on three different regions (dotted arrows) within the cortical thalamic system: 3 ROI are structurally (forward and backward) connected (solid arrows). Model A: GSW as autonomous input on the thalamus; Model B: GSW as autonomous input on the ventromedial prefrontal cortex (vmPFC). Model C: GSW as autonomous input on the precuneus (BA 7). GSW: Generalised Spike and Wave discharges; BA: Brodmann Area.

We applied DCM to EEG-correlated fMRI data to understand the dynamic interaction between brain regions known to be involved in the initiation and cessation of GSW discharges and with a brain region known to be related to conscious awareness, the precuneus. We compared a family of models of effective connectivity focusing on a set of cortical regions and the thalamus. We tested and compared the following models in relation to the GSW discharges: when treated as autonomous input GSW activity enters the cortico-thalamic loop: 1. via the thalamus (following the centrencephalic theory); 2. via the ventromedial prefrontal cortex (vmPFC) (following the cortical focus theory); or 3. via the precuneus (i.e. the state of the precuneus – a key region for the sustenance of consciousness - gates pathological activity via the cortico-thalamic network); see Fig. 1 for an illustration of the models.

## Materials and Methods

### Patients

In order to apply DCM analysis, we re-analysed the resting-state EEG-fMRI data of 32 IGE patients studied previously [23]. Ten patients from the original population did not have any GSW discharges during the 35-min fMRI session and were hence discarded. The EEG/fMRI data of the remaining 22 patients were pre-processed and analysed using SPM8b. In 15 cases no GSW-related BOLD changes were found in the thalamus (14 cases) and in the precuneus (one case), leaving data from 7 patients (5 females) in whom significant GSW-related BOLD signal changes (increase and/or decrease) were revealed in all three regions of interest: the thalamus, vmPFC (Brodmann Area 10) and precuneus (Brodmann Area 7). The 7 selected patients represent all the cases that satisfied the necessary criteria for the DCM analysis; See table S1 in the Supplementary Materials for a description of the cases that did not meet the criteria. According to the ILAE 1989 classification scheme [40] five patients were affected by Juvenile Absence Epilepsy (JAE) (patients #2a, 5, 7, 9a, 11 in [23]) and two patients by Juvenile Myoclonic Epilepsy (JME) (patients #18, 21a in [23] (Table 1). Structural MRI was normal in all patients.

**Table 1:** Clinical details of patients studied based on ILAE diagnostic categories.

Id no.	Age/Gender	Epilepsy syndrome/Syndrome subtype	Seizure type frequency (age onset/yrs)	Therapy
2a	24/F	IGE/JAE	abs 15/d (10), GTCS	LTG,ESM, CBZ,TPM
5	43/F	IGE/JAE	abs daily (8) , GTCS 4/yr (13)	VPA,LTG,CLB
7	22/F	IGE/JAE	abs 2–3/d (8), GTCS 3/mth (19)	LEV, ESM, TPM
9a	18/M	IGE/JAE	abs weekly (15)	nil
11	33/F	IGE/JAE	abs daily (teens)	VPA, LTG
18	20/M	IGE/JME	abs 3/d (<10), GTCS 1/mth (13), MJ(teens)	LEV
21a	37/F	IGE/JME	abs 10 d (7) GTCS 2/yr (12), MJ teens	VPA, GBP, CLB

ID no: patient identification as in Hamandi et al., 2006, table 1.

JAE, juvenile absence epilepsy; JME, juvenile myoclonic epilepsy; abs, absence seizures; MJ, myoclonic jerks; GTCS, generalized tonic clonic seizures; CBZ, carbamazepine; CLB, clobazam;; ESM, ethosuximide; GBP, gabapentin; LEV, leviteracetam; LTG, lamotrigine; TPM, topiramate; VPA, sodium valproate; M, male; F, female; d, day; wk, week; mth, month; yr, year.

a patients studied in two successive sessions.

### EEG-fMRI acquisition and analysis

The methods pertaining to data acquisition are described elsewhere [23]. In brief, ten-channel EEG was recorded using MR-compatible equipment, at Fp1/Fp2, F7/F8, T3/T4, T5/T6, O1/O2, Fz (ground) and Pz as the reference [10–20 system), along with bipolar electrocardiogram [41]. Seven hundred and four T2*-weighted single-shot gradient-echo echo-planar images (EPI; TE/TR: 40/3000, 21 interleaved axial slices of 5 mm thickness, acquired continuously and parallel to the inter-commissural line, FOV 24×24 cm^2^, 64×64 matrix) were acquired over a 35-min session on a 1.5 Tesla Horizon EchoSpeed MRI scanner (General Electric, Milwaukee, WI). Patients were asked to rest with their eyes closed and to keep still.

FMRI data were processed and analysed using SPM8b (http://www.fil.ion.ucl.ac.uk/spm/).

After discarding the first four image volumes, the EPI time series were realigned and spatially smoothed with a cubic Gaussian Kernel of 8 mm full width at half maximum and normalised to MNI space.

A general linear model (GLM) was constructed to assess the presence of regional GSW-related BOLD signal changes. GSW events were represented as variable-duration blocks beginning at the onset of GSW as identified on the MR-synchronised EEG by two expert observers (AEV and RT) and ending upon GSW cessation.

Motion-related effects were modelled in the GLM by 24 regressors of the 6 scan realignment parameters and a Volterra expansion of these [42], plus scan nulling Heaviside terms for large (inter-scan displacement>0.2 mm) motion effects [43], [44]. No global scaling was employed. In addition, confounds were included to account for cardiac-related signal changes [45].

The GSW event blocks were convolved with the canonical hemodynamic response function (HRF), and its temporal and dispersion derivatives, to form regressors testing for GSW-related BOLD signal changes. Significant positive and negative BOLD signal changes correlated with GSW were identified by means of an F-contrast across the three regressors of interest and recorded as activation and deactivation depending on the response shape. The resulting SPMs were thresholded at p<0.001 [46] to define regions of interest (inference on these regional effects using multiple comparison correction are reported in [23]).

### Effective connectivity

The DCM analysis was performed for three ROI: thalamus, vmPFC, precuneus. For all ROI we used spherical volumes with a 5 mm radius. For ROI selection within the thalamus we chose the axial slice that showed the largest cluster and placed the ROI so as to cover the region. In patients with bilateral thalamus involvement, we selected only one ROI, on the side of the largest cluster. For ROI selection within vmPFC, we placed the ROI in the axial slice within the Brodmann Area 10 and the side containing the largest area of signal BOLD change. The precuneus ROI was placed within the medial sagittal slice, rostrally to the middle of the parieto-occipital sulcus. In patients showing bilateral precuneus involvement, we placed the ROI on the side of the largest cluster. The ROI positions were defined using Talairach Daemon, http://ric.uthscsa.edu/project/talairachdaemon.html); the Talairach coordinates and equivalent Z-scores of the selected regions are listed in Table 2. Following the standard DCM procedure in SPM, a summary time series was derived for each ROI by computing the first principal eigenvariate of all super-threshold voxel time series within the ROI.

**Table 2:** DCM Regions of interest.

Id no.	Talairach coordinates of ROIs			Cluster size (voxels)/Z-score			
	Thalamus	vmPFC	Precuneus	Thalamus	vmPFC	Precuneus	
2a	x = 4 y = −16 z = 2	x = −24 y = 62 z = 14	x = −22 y = −76 z = 50	19/3.42	81/5.55		81/4.27
5	x = −2 y = −24 z = 4	x = 26 y = 46 z = 26	x = 14 y = −80 z = 4	81/4.3	73/3.78		81/4.90
7	x = 10 y = −12 z = 8	x = 30 y = 42 z = 22	x = 8 y = −54 z = 64	81/7.05	81/7.07		81/>7.53
9a	x = 2 y = −16 z = 0	x = 12 y = 52 z =	x = 10 y = −58 z = 20	14/3.24	81/5.28		81/5.64
11	x = 4 y = −14 z = 0	x = −2 y = 66 z = 24	x = −4 y = −70 z = 40	78/4.13	81/3.98		81/4.74
18	x = −16 y = −14 z = 2	x = −24 y = 46 z = 14	x = 16 y = −72 z = 40	81/7.29	81/6.07		78/4.58
21a	x = 8 y = −18 z = 8	x = 44 y = 56 z = 4	x = 0 y = −72 z = 52	81/>7.53	64/7.53		61/5.60

ID no: patient identification as in Hamandi et al., 2006, table 1.

vmPFC: Ventromedial Prefrontal Cortex. Talairach Coordinates of the ROIs selected (obtained using Talairach Daemon, http://ric.uthscsa.edu/project/talairachdaemon.html); Z-scores are reported for local voxel maxima.

The regional responses were filtered, whitened and the nuisance effects (motion, cardiac) were subtracted to leave only GSW-related effects. To account for the effect of scan nulling of large motion events (which effectively removes any signal change in the affected volumes) [44], the GSW epoch was removed when it occurred during these motion-laden periods. The net effect of this procedure was to remove any events associated with large-scale head motion from consideration within the DCM.

Using the DCM module as implemented in SPM8b three linear models were constructed. Each comprised the three ROI as reciprocally (forward and backward) connected regions and GSW event blocks considered as autonomous input to each of the three regions, one at a time a) GSW as autonomous input on the thalamus (Model A), b) GSW as autonomous input on vmPFC (Model B), c) GSW as autonomous input on the precuneus (Model C). Hence, three models were evaluated per subject (see the schematic in Fig. 1).

After the estimation of parameters of each competing model, they were compared using Bayesian Model Comparison (BMC) where the evidence of each model, computed from estimated parameters distributions, is used to quantify the model plausibility [36], [39]. Given two models *m_1_* and *m_2_*, one can compare them by computing the difference in their log-evidence ln *p*(*y | m_1_*)- ln *p*(*y | m_2_*). If this difference is greater than about 3 (i.e. the relative likelihood is greater than 20∶1) then one asserts that there is strong evidence in favour of one of the models. This is commonly calculated based on the *F* value of each model, which is the negative marginal log-likelihood or negative log-evidence: *F* = −ln *p*(*y | m*). For more details about BMC, see [47], [48].

Assuming that data from each subject are conditionally independent, the evidence at the group level is obtained by multiplying the marginal likelihood, or, equivalently, by adding the log-evidences from each subject [47].

## Results

### GSW-related BOLD patterns

**Figure 2:**
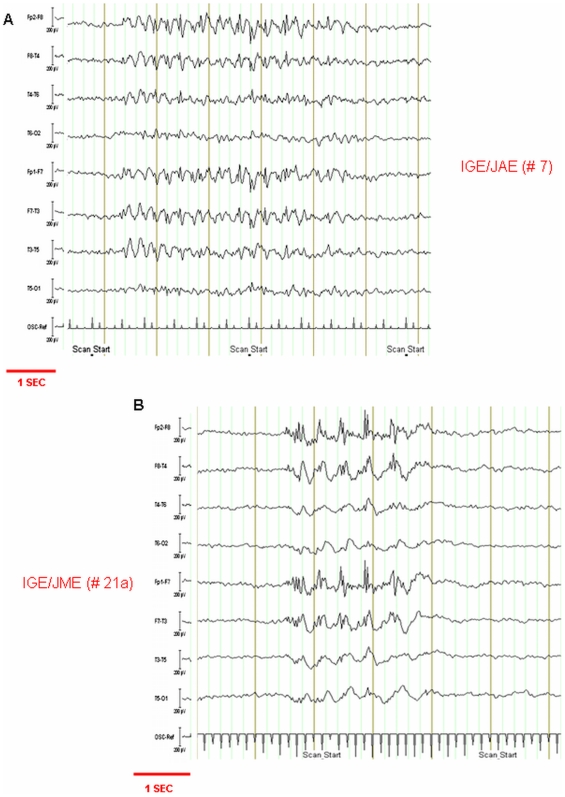
Representative example of EEGs recorded during scanning after scanning artefact subtraction. The EEG traces were analysed following pulse (not shown) and image artifact subtraction; EEG traces are displayed as bipolar montage. OSC: scanner slice pulse used for EEG artifact correction, and EEG-fMRI synchronization (7/s). (A) IGE/JAE: patient (ID #7). The trace shows an epoch of 3.5 Hz generalised spike-wave complexes (length ∼4 seconds) with anterior predominance. (B): IGE/JME: patient (ID #21a).The trace shows an epoch of 2.5−3 Hz generalised multispike-wave complexes (length ∼2.5 seconds) with anterior predominance.

**Figure 3:**
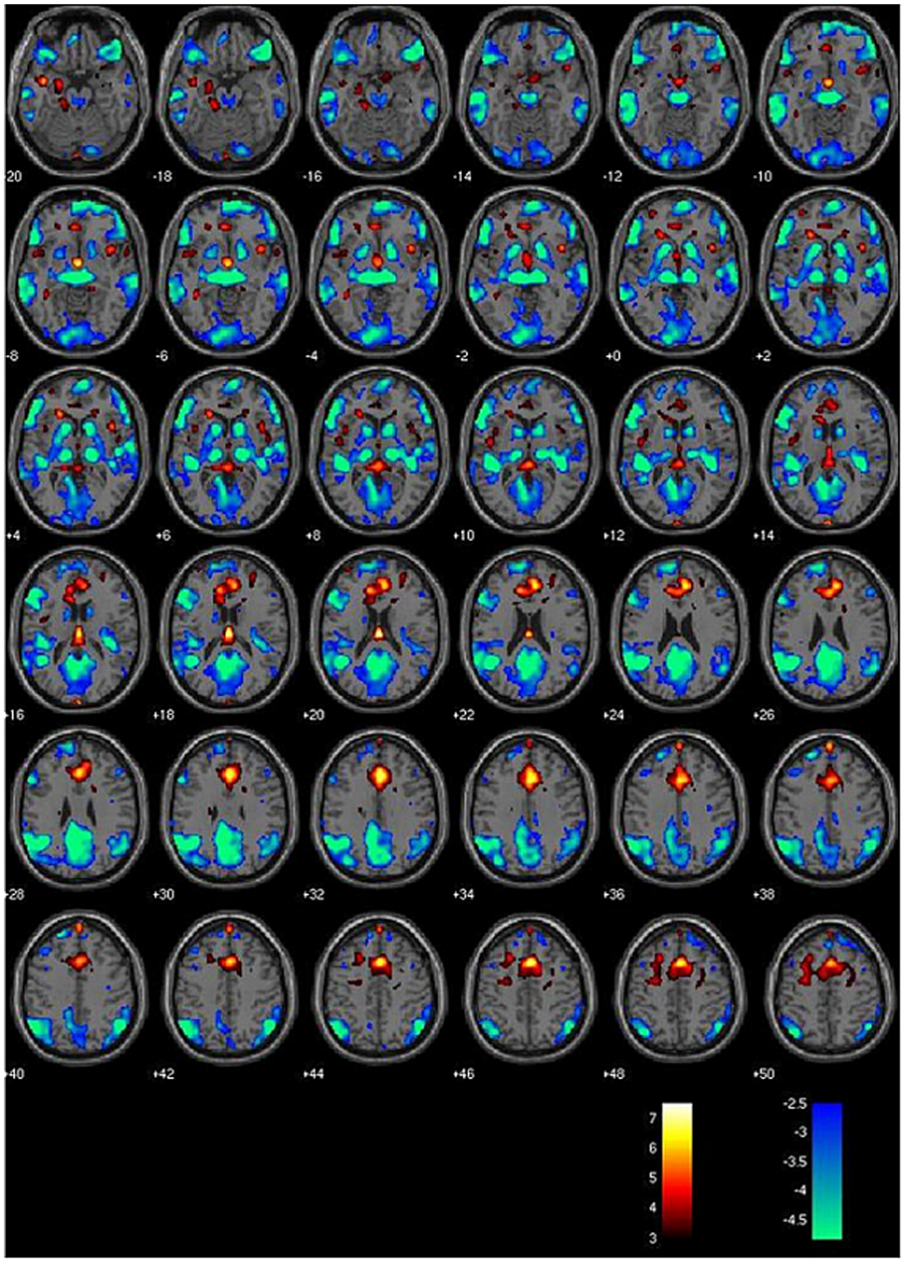
EEG-FMRI statistical parametric map in a patient with JME. A colour-coded overlay of SPM{t} (red: positive BOLD response; green: negative BOLD response) (p<0.05 corrected for Family-Wise Error-FWE) onto the slices overlay shows, BOLD signal increase in bilateral cingulated gyrus (BA32) and BOLD signal decrease in bilateral thalamic, bilateral caudate, right medial frontal gyrus (BA10), left superior temporal gyrus (BA39), right precuneus (BA7), bilateral inferior parietal lobuli (BA39). Clusters labelling according to Talairach Daemon, (http://ric.uthscsa.edu/project/talairachdaemon.html). BA: Brodmann Area

Good quality EEG was obtained following pulse and gradient artefact subtraction, allowing reliable identification of epileptiform discharges (see Fig. 2). EEG discharge features are summarized in table 3. The reader should refer to table 3 and [23] for the detailed patterns of the GSW-related BOLD signal changes in each patient. Figure 3 shows a representative example of a BOLD map for one patient with JME (#21a).

**Table 3:** EEG-fMRI results.

Id no./Epilepsy syndrome	No. of GSW events	Duration of GSW events, median (range) (seconds)	EEG-fMRI results for DCM ROIs			
			Thalamus		vmPFC	Precuneus
2a/JAE	3	7.3 (4.4−7.7)	B **(i)**	B (>L) **(d)**		L **(d)**
5/JAE	18	0.6 (0.4−3.6)	B(>L) **(i)**	R **(i)**		B **(i)**
7/JAE	2	4.3 (3.4−5.3)	B(>R) **(i)**	B **(i)**		B **(i)**
9a/JAE	8	1.9 (0.7−3.6)	R **(i)**	R **(d)**		B **(d)**
11/JAE	189	1.6 (0.3−73.9)	B(>L) **(i)**	B **(d)**		B **(d)**
18/JME	25	1.3 (0.4−3.4)	B(>L) **(i)**	B(>L) **(i)**		B **(i)**
21a/JME	60	1.4 (0.4−8.4)	B **(d)**	R **(d)**		B **(d)**

ID no: patient identification as in Hamandi et al., 2006, table 1. Summary of results for all EEG-fMRI sessions: number and duration of GSW epochs, regions of BOLD signal change labelled in accordance with direction of HRF loading, vmPFC: Ventromedial Prefrontal Cortex;

**(i)** BOLD signal increase;

**(d)** BOLD signal decrease; B: bilateral, L: left, R: right. All SPMs corrected for multiple comparisons using random field theory (p<0.001, patients 2a, 7, 9a, 18; p<0.05, patients 5, 11, 21a).

In accordance with the selection criteria, significant GSW-associated BOLD signal changes were found in the thalamus, in the frontal lobe limited to the vmPFC, and precuneus (see table 3 and [23]). Frontal cortex and precuneus showed a positive BOLD response in 3 patients (2 JAE and 1 JME) and a negative BOLD response in the remaining 4 patients (3 JAE and 1 JME). In two cases (#2a, #9a) our results were different from Hamandi's previous single-subject analysis results when fewer confounds were included (no global scaling and 6 scan realignment parameters with their first order expansion in [23]). We note that Hamandi et al., showed a consistent pattern of thalamic signal increase and a cortical signal decrease which involved the precuneus and prefrontal cortex.

### Effective connectivity

**Figure 4:**
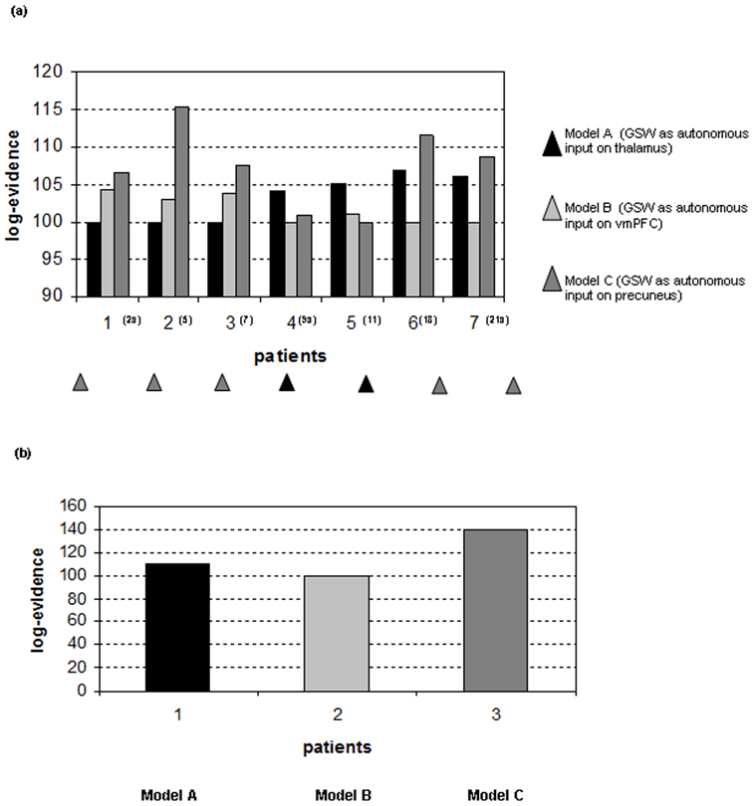
Effective connectivity model comparison results. Bayesian Model Selection (BMC) among DCMs for the three models tested. (a): differences between log-evidences for model A, B, C for each subject. The triangles identify the best model on the basis of the subject's highest log-evidence difference. A difference greater than 3 is highly significant. For illustration purposes we added a constant value of 100 to all log-evidence differences. The numbers inside brackets (on the x-axis) correspond to ID no in Hamandi et al. [23]. (b): graph showing the difference between the log-evidence at the group level, i.e. pooled over subjects, for the three models. For illustration purposes we added a constant value of 100 to all log-evidence differences.

Figure 4a shows the log-evidence for the three models, in each subject. Bayesian Model Comparison (BMC) identified model C (GSW immediately influences the precuneus) to be the best in 5 cases. In patients #5, #7, #18 model C was found to be significantly more likely than both models A (GSW immediately influences the thalamus) and B (GSW immediately influences the vmPFC), whereas in patient #2a it was significantly better than model A and in patient #21a better than model B only. In the remaining two cases (#11, #9a) model A was better than models B and C. Interestingly model B was never significantly better than model C and in only one subject (#7) it was more likely than model A. Table 4 shows the *F* values (i.e. the negative log-evidence) in absolute numbers. Figure 4b shows the log-evidence for the three models at the group level. Models C and A are clearly more likely than model B and over patients, there is strong evidence in favour of model C over model A and B.

**Table 4:** DCM F values.

Id n°	Model A	Model B	Model C
2a	F = −3427.2898	F = −3422.9665	F = −3420.6739
5	F = −3039.0606	F = −3036.7609	F = −3023.5675
7	F = −1762.445	F = −1758.6259	F = −1754.9096
9a	F = −2972.7801	F = −2976.9679	F = −2976.0056
11	F = −3089.2781	F = −3093.2254	F = −3094.3386
18	F = −3210.5648	F = −3217.2931	F = −3205.6735
21a	F = −2377.5158	F = −2383.6488	F = −2374.9833

ID no: patient identification as in Hamandi et al., 2006, table 1.

F value (i.e. the negative log-evidence) for each model at single subject level analysis. See the text for details.

Assuming that all patients in the group are representative of IGE we generalized the results of group analysis to the population level. The *p* value calculated for each model were extremely close to zero for models B and A, and close to 1 for model C, demonstrating the latter to be very likely at the population level.

## Discussion

We investigated the causal relationship between neuronal activity as reflected by BOLD signals in three brain regions, namely the thalamus, the vmPFC and the precuneus, in relation to the onset and offset of GSW in 7 patients affected by IGE. The thalamus and the frontal cortex are key structures in well established hypotheses on GSW generation [29]–[31], [49], [50]. The inclusion of the vmPFC was motivated by recent evidence for its primary role in the generation of GSW in absences [33], [34], [51] and by the frequent observation of its prominent BOLD increase and/or decrease compared to other frontal cortical areas in EEG-fMRI studies [22]–[25]. Our results are consistent with the precuneus, as a key region changing its activity with altered states of vigilance, influencing the occurrence of generalized seizures. The precuneus has direct connections with the frontal lobe (prefrontal cortex) [52]–[54] and thalamus [55]–[57].

Applying DCM to fMRI data simultaneously acquired with EEG we found that, for the models tested, in five out of seven patients studied, electroencephalographic discharges first affected the precuneus. This finding became more evident at the group and population level so that the evidence in favour of model C (GSW as autonomous input to precuneus) was significantly higher than for models A (GSW as autonomous input to thalamus) and B (GSW as autonomous input to vmPFC). In the remaining two cases, BMC showed model A to be the best. The discrepancy between the results of the analysis of the single subjects is not unexpected. We note that in one of the pioneer DCM studies on evoked potentials, Garrido et al [47] found reproducible results in seven out of eleven patients (in whom one of the models tested was the best over subjects) whereas the consistency of their conclusion is more evident at the group level. In our study, the consistency of the results at both the individual and group level is similar.

Our finding, that GSW onset and offset are more directly linked to the neural activity (as reflected by the BOLD signal) in the precuneus than in the other tested regions, implies a dependency of the cortico-thalamic loop on the precuneus and hence its state, i.e. a causal link. A possible interpretation is that changes in the precuneus state (as increase or decrease of it neuronal activity), which reflects spontaneous fluctuations in awareness, act on the thalamic-(frontal) cortical network facilitating the development of GSW. This is in contra-distinction to previous suggestions [22]–[24] that decreases in precuneal activity reflect the semiology of impaired consciousness and are a consequence of GSW.

A similar hypothesis has been already proposed by Archer et al [58], who observed a significant posterior cingulate negative BOLD response in 5 IGE patients with interictal GSW discharges whereas no BOLD signal changes were detected in thalamus or prefrontal cortex. The authors suggested that decrease in the posterior cingulate activity and associated regions may be involved in initiation of GSW activity.

Additionally, an fMRI study showed BOLD signal decrease in the posterior cingulate in IGE subjects following photic stimulation whether or not GSW occurred, while control subjects showed no change in this region [59]. Such changes would be consistent with decrease in the posterior cingulate activity being a precursor (or facilitator) of GSW, rather than being a secondary phenomenon. The posterior cingulate cortex is adjacent to precuneus and some authors proposed it as part of precuneal cortical area [60], [15].

### Precuneus, awareness and GSW generation

According to the current thinking of the pathophysiology of GSW, there are two prerequisites for the occurrence of this pathological activity: 1) the pathological thalamo-(frontal) cortical interactions and 2) the so-called mild diffuse epileptogenic state of the cortex [ictogenicity of the cortex;61]–[64]. Our findings suggest that changes in the level of awareness and hence precuneal activity, may increase the likelihood of an epileptogenic cortical state to arise and GSW to be generated within the thalamo-(frontal) cortical network.

The precuneus' neuronal state, and hence the level of awareness, may, consequently, reflect a “physiological initiator” of generalized synchronous discharges. The existence of a transient facilitating state of the brain which increases cerebral susceptibility to GSW generation has been recently demonstrated in patients with absences, by synchronization measures and MEG source imaging methods [65]. This “idle” state is not part of the ictal process itself but allows vulnerable regions to generate epileptic discharges; electrically, it reflects a long-range de-synchronization between cortical sources which takes place few seconds before seizure onset. Fluctuations in the level of awareness, and hence in precuneal activity, could account for this facilitating effect. It is well established that the probability of absence occurrence depends on the level of awareness of the patient. Guey and colleagues, in 1969 [4], identified factors influencing the occurrence of epileptic paroxysms in 30 patients with absences; particularly they found that, when an epileptic patient focused his attention, the likelihood of GSW discharges diminished considerably. Moreover, inactivity or the moment of rest after an accomplished task or monotonous task can be regarded as factors favouring the occurrence of paroxysms. The fact that these processes can be detected also at the observational level implies a time scale of these changes of the order of seconds. This, in turn, makes fMRI a suitable tool to reveal such phenomena. A recent GSW-correlated EEG-fMRI study, indeed, demonstrated BOLD signal and hence neuronal activity changes - including within the precuneus - to occur several seconds before the appearance of the pathological GSW activity on scalp EEG [66]. Moreover, the application of DCM to fMRI data from animal models with absences [39] has been validated with intracranial recordings.

Previous EEG-fMRI studies showed BOLD signal decrease in the DMN in relation to GSW apparently not correlated with clinical manifestations [23], [24]. This also argues for the proposed permissive role of precuneal activity changes contributing to an epileptogenic state and eventually GSW generation rather than DMN changes being a consequence of the semiology of impaired consciousness. A common pattern of negative BOLD responses in the DMN was observed in relation to focal interictal spikes in patients with temporal lobe epilepsy [67]. It remains to be seen whether precuneal activity plays a similarly permissive role in patients with focal epilepsy and complex partial seizures, i.e. periods of impaired consciousness.

### EEG-fMRI adds to electrophysiological data on GSW generation and precuneal involvement

While there is little evidence of a strict consequentiality between a particular state of vigilance and the occurrence of GSW discharges, there is a notable lack of studies focusing on the possible role of cortical structures (particularly the precuneus) other than the thalamus and frontal cortex in GSW. However, evidence from scalp EEG source reconstruction analysis suggests that the precuneus participates in the generation of fast sleep spindles at 14 Hz [68]. Animal models of absence epilepsy showed the possibility of a transition between sleep spindles and GSW suggesting a common origin [29], [69]. According to this hypothesis, the circuit which normally generates sleep spindles leads to GSW under the condition of a cortical hyper-excitability [1]. The existence of an active cortical spindle source located in the region of the precuneus is in line with our findings of its involvement in GSW discharges. Additionally, source reconstruction of the interictal spontaneous EEG activity has shown elevated bilateral parieto-occipital cortex involvement in patients with IGE compared to healthy subjects [65]. However, as noted previously, we are not aware of the precuneus having been identified in relation to GSW generation in neither EEG nor MEG.

In contrast to surface electrophysiological recordings, fMRI studies with concurrent EEG in patients with GSW discharges have shown common significant haemodynamic changes not only in the thalamus and frontal cortex, but also in the precuneus and other brain regions (encompassing fronto-parietal association cortices) of the DMN [20]–[22], [70].

FMRI's relatively homogeneous sensitivity across the brain relative to that of scalp EEG may explain why recent EEG-fMRI studies have been able to reveal precuneal involvement in epilepsies characterized by impaired consciousness and in particular associated with GSW [22], [24].

The application of connectivity analysis techniques based on fMRI data may improve our understanding of the interactions between brain regions haemodynamically involved during GSW discharges. David et al., applied DCM analysis to fMRI data acquired in an animal model of absences [39]. They compared alternative neural models including the thalamus, the striatum and the somatosensory cortex, in which each region is modelled in turn as the driver of the pathological activity and showed that the somatosensory cortex to be the more likely driver. This is line with previous findings in animal models [30], [31]. Despite the presence of significance symmetrical activated clusters in retrosplenial cortex the authors assumed the posterior cortical regions to be involved only during the spreading of the pathological activity (downstream effect) and therefore did not include them in their models. No inference can be made on the putative role of regions not included in the models. We note that retrosplenial cortex, together with precuneus and posterior cingulate cortex, has been shown to be a critical node in the network correlates with consciousness in humans and animals [71].

### Methodological Considerations

Brain connectivity based on fMRI data, can be investigated via two different approaches: functional connectivity and effective connectivity analysis. Functional connectivity is data-driven and assesses statistical dependencies between fMRI signals from different brain regions without consideration of the underlying neuronal activity. In contrast, analyses of effective connectivity test hypotheses based on modelling of neuronal activity and a forward model describing how this activity is translated into the fMRI signals [72]. DCM of fMRI can thus provide information about the directionality of functional relations between positive or negative BOLD clusters and is context-dependent [72], [73]. Furthermore, our choice of effective connectivity approach rather than a data-driven functional connectivity analysis was motivated by the following considerations: 1) we had clear hypotheses to be tested about the GSW pathophysiology, and 2) the capacity of effective connectivity analyses to determine causality of the interactions between a set of brain regions makes it much more powerful.

There are different approaches for modelling effective connectivity from functional MRI, which include structural equation modelling (SEM), vector-autoregression models and DCM [35]. DCM represents a departure from other existing approaches since it assumes that the responses are driven by known or measurable regional changes that may be controlled experimentally [35]. An important conceptual aspect of DCM for neuroimaging pertains to how experimental or known effects enter the model and cause neuronal responses; designed experimental inputs may elicit responses through direct influences on specific anatomical nodes (driving or autonomous inputs), or they may affect the system by inducing changes in coupling among brain areas (modulatory inputs). In the context of intrinsic brain activity, and in particular brain states defined in relation to paroxysmal discharges such as GSW, the notion of experimental effects requires re-interpretation. In our approach, the GSW epochs, represented as blocks, correspond to the onset and offset of a perturbation of endogenous neuronal activity in the postulated network and epileptic activity is supposed to act as an endogenous autonomous effect, since it can influence directly the neuronal state of the specified anatomical nodes. There is good evidence of the validity of this approach in relation to intra-cerebral electrophysiology in rats [39]. Our family of models is designed to compare the causal hierarchy between the three postulated regions of interest, and representing GSW as an autonomous effect must be seen as meeting a requirement of DCM with respect to this aim. We could have increased the number of cases included in the analysis by relaxing the requirement for involvement of the three regions of interest, for example cases that do not show significant GSW-related BOLD changes in the thalamus; however, we believe that this would have undermined the credibility of the model comparison due to their known importance in GSW generation. Furthermore, in contrast to DCM for M/EEG [74] DCM for fMRI is limited to the comparison of models with an identical numbers of nodes. This is because in Bayesian model comparison, the models can be different but the data must be the same. In fMRI the data are derived from the nodes whereas in M/EEG, the data are taken from the sensors.

The causal link revealed in our study (i.e. precuneus activity facilitates GSW) is limited to onset and offset of GSW discharges. Therefore, our findings do not preclude a reverse causal relationship in which GSW accompanied by impairment of consciousness leads to (further) deactivation of the precuneus. This could be addressed by studying ictal GSW data using a similar methodological approach.

In common with all DCM-based inferences, our conclusions are valid solely with respect to the family of tested models; there may be brain areas which are involved in the GSW generation processes that were overlooked because of their apparent lack of haemodynamic involvement.

### Conclusion

In this study we have demonstrated an active role in generalised epilepsy for the precuneus, a region previously neglected in electrophysiological studies of GSW. Using Dynamic Causal Modelling based on EEG-fMRI data we showed that the precuneus not only is strongly connected with the frontal cortex and the thalamus but also that the neuronal activity in this area may facilitate epileptic activity within a thalamo-cortical loop, the existence of which is well established. These findings suggest that GSW may arise through the direct influence of the neuronal state of the precuneus associated with spontaneous changes in the level of awareness.

## Supporting Information

Table S1 Description of fMRI results for the cases that did not satisfy the selection criteria for the DCM analysis. Summary of results for all cases for which GSW activity was captured during EEG-fMRI but did not meet the selection criteria for the DCM analysis (extracted from [23]). All SPMs corrected for multiple comparisons using random field theory (p<0.05). JAE: Juvenile Absence Epilepsy; JME: Juvenile Myoclonic Epilepsy; IGE-GTCS: epilepsy with generalized tonic clonic seizures only; CAE: childhood absence epilepsy. Id no: patient identification number. Direction of BOLD change: ↑ - increase, ↓ - decrease, ↕ - biphasic. B - bilateral, L - left, R - right, m - global maximum, BS - brainstem, cereb - cerebellum, temp - temporal lobes, occ - occipital lobes, ss - sagittal sinus (draining vein).
(0.06 MB DOC)
