# Supplementary material for: Causal Hierarchy within the Thalamo-Cortical Network in Spike and Wave Discharges
Source: PLoS One. 2009 Aug 3;4(8):e6475. doi: 10.1371/journal.pone.0006475 (PMC2715100; doi:10.1371/journal.pone.0006475)
Supplement: Table S1 — Description of fMRI results for the cases that did not satisfy the selection criteria for the DCM analysis. Summary of results for all cases for which GSW activity was captured during EEG-fMRI but did not meet the selection criteria for the DCM analysis (extracted from [23]). All SPMs corrected for multiple comparisons using random field theory (p<0.05). JAE: Juvenile Absence Epilepsy; JME: Juvenile Myoclonic Epilepsy; IGE-GTCS: epilepsy with generalized tonic clonic seizures only; CAE: childhood absence epilepsy. Id no: patient identification number. Direction of BOLD change: ↑ - increase, ↓ - decrease, ↕ - biphasic. B - bilateral, L - left, R - right, m - global maximum, BS - brainstem, cereb - cerebellum, temp - temporal lobes, occ - occipital lobes, ss - sagittal sinus (draining vein). (0.06 MB DOC) [file pone.0006475.s001.doc]

**Supplementary Material**

**Table S1. Description of fMRI results for the cases that did not satisfy the selection criteria for the DCM analysis.**

| **Diagnostic category** | **Id**  **No.** | **Regions of significant BOLD signal change** | | | | |
| --- | --- | --- | --- | --- | --- | --- |
|  |  | **Thalamus** | **Frontal** | **Parietal** | **Posterior**  **cingulate / precuneus** | **Other** |
| **JAE** | 1 | - | ↑ B (L **m)** | ↓ B | ↑B | ↑ ss, cereb, temp |
|  | 4 | - | - | - | - | - |
|  | 6 | - | - | - | - | - |
|  | 8 | - | ↓ B (L **m**) | ↓ B. | ↓ B | - |
|  | 10 | - | - | - | - | - |
| **JME** | 15 | - | - | - | - | ↓ occ **m**,↕ R BS |
|  | 17 | - | - | - | - | - |
|  | 19 | - | - | - | - | - |
|  | 20 | - | - | - | - | - |
|  | 22 | - | ↓ B | ↓ B (L **m**) | ↓ B | - |
| **IGE-GTCS** | 24 | - | - | - | - | - |
|  | 25 | - | ↓ B | ↓ B (L **m**) | ↓ B | - |
|  | 26 | - | - | - | - | - |
| **CAE** | 32 | - | ↓ B | ↓ B | ↓ B | ↑ cereb **m** |

Table S1. Summary of results for all cases for which GSW activity was captured during EEG-fMRI but did not meet the selection criteria for the DCM analysis (extracted from [23]). All SPMs corrected for multiple comparisons using random field theory (p<0.05). JAE: Juvenile Absence Epilepsy; JME: Juvenile Myoclonic Epilepsy; IGE-GTCS: epilepsy with generalized tonic clonic seizures only; CAE: childhood absence epilepsy. Id no: patient identification number. Direction of BOLD change: ↑ - increase, ↓ - decrease, ↕ - biphasic. B - bilateral, L - left, R - right, **m -** global maximum, BS - brainstem, cereb – cerebellum, temp - temporal lobes, occ - occipital lobes, ss - sagittal sinus (draining vein).
